# Early onset, multiple, bilateral fibroadenomas of the breast: a case report

**DOI:** 10.1186/s12905-021-01311-7

**Published:** 2021-04-21

**Authors:** Cecilia J. Im, Ashlie Miller, Ronald Balassanian, Rita A. Mukhtar

**Affiliations:** 1 School of Medicine, University of California San Francisco, San Francisco, CA USA; 2 Department of Cancer Genetics, University of California San Francisco, San Francisco, CA USA; 3 Department of Pathology, University of California San Francisco, San Francisco, CA USA; 4 Department of Surgery, Division of Surgical Oncology, University of California San Francisco, San Francisco, CA USA

**Keywords:** PTEN, Fibroadenoma, Genetic testing, Case report

## Abstract

**Background:**

While fibroadenomas are common in the general population, affecting 10–20% of women, they are rarely early-onset, multiple, and bilateral.

**Case presentation:**

An 18-year-old woman presented with a 6 year history of multiple, bilateral breast masses without family history of breast disease. Magnetic resonance imaging (MRI, Fig. 1) of the breasts showed innumerable, bilateral breast masses ranging in size from 0.5 to 4 cm. Two needle biopsies showed fibroadenoma. Although the patient’s family history did not meet National Comprehensive Cancer Network (NCCN) guidelines for genetic testing, it was performed due to the rarity of her presentation. Genetic testing identified a pathogenic mutation in the phosphatase and tensin homolog (*PTEN*) gene.

**Conclusions:**

A germline mutation in *PTEN* is associated with an increased risk of breast cancer and often occurs as part of Cowden Syndrome. This case highlights the importance of genetic testing in patients with unusual presentations of early-onset, bilateral, and multiple (greater than four) fibroadenomas.

**Supplementary Information:**

The online version contains supplementary material available at 10.1186/s12905-021-01311-7.

## Background

The *PTEN* protein acts as an antagonist to the P13k/Akt pathway. Insufficient suppression in the setting of a *PTEN* mutation leads to increased cell proliferation with decreased apoptosis [1]. PTEN hamartoma tumor syndrome is a family of clinical syndromes characterized by germline mutations in the *PTEN* gene [2]. The best characterized of these is Cowden Syndrome which is associated with increased lifetime cumulative risk of developing cancer in the breast (67–85%), thyroid (35%), kidney (33%), and endometrium (28%) [3]. Additionally, benign breast disease is common in Cowden syndrome, with fibroadenomas found in approximately 35% of *PTEN* mutation carriers. These fibroadenomas are more likely to be complex and hyalinize at an early age [4].

While fibroadenomas are common in the general population, affecting 10–20% of women, they are rarely multiple and bilateral [5]. Among women with fibroadenomas, only 15% have 2–4 masses in one breast and only 11% have bilateral masses [6]. Additionally, fibroadenomas in adolescents are rare, found in just 0.5 to 4% of patients [7]. The reported data regarding women with bilateral and multiple fibroadenomas is currently limited to less than 20 case reviews without genetic data. Only one other case report characterizes an adolescent with bilateral and more than 4 masses but this study lacks genetic data [8].

Current NCCN guidelines state that major criteria for genetic testing for PTEN hamartoma tumor syndrome include breast, endometrial, or follicular thyroid cancer, multiple gastrointestinal hamartomas, macrocephaly, and the presence of mucocutaneous lesions including trichilemmoma, palmoplantar keratoses, or extensive oral mucosal papillomatosis [9]. This case report provides evidence for obtaining germline genetic testing in patients with unusual presentations of fibroadenomas.

## Case presentation

**Fig. 1:**
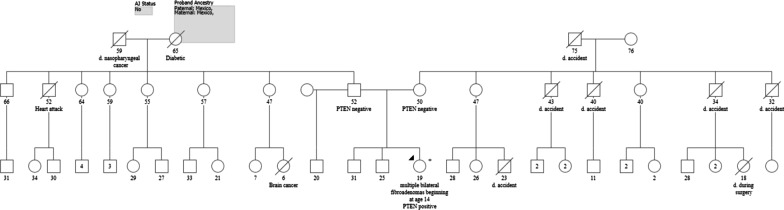
The family pedigree was significant for nasopharyngeal cancer in a paternal grandfather and brain cancer in a paternal cousin

An 18-year-old woman presented with multiple, bilateral breast masses with cyclical discomfort associated with her menses. The first mass developed at age 12 near menarche, with several more masses developing and increasing in size over time. She was otherwise healthy, and family history was notable for nasopharyngeal cancer in her paternal grandfather and brain cancer in a paternal cousin who died at age 6 (Fig. 1).

On physical examination, the breast tissue was nearly replaced by multiple smooth, mobile, non-tender, well circumscribed nodules each measuring 1–2 cm in size, with a 4 cm mass in the outer right breast. She had no lymphadenopathy, trichilemmomas, oral papillomatosis, or acral keratosis. There was keratosis pilaris of the upper arms.

**Fig. 2:**
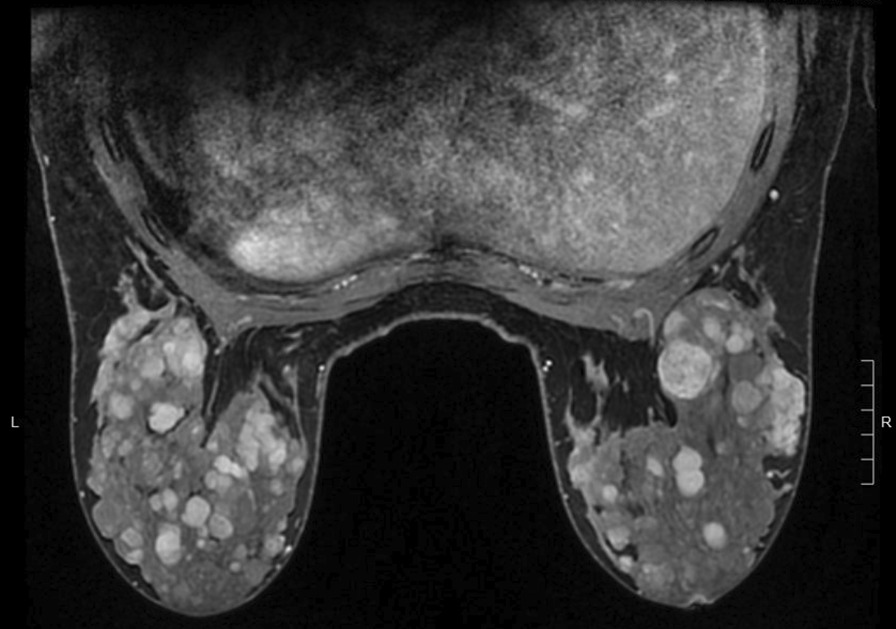
Contrast enhanced breast MRI shows innumerable bilateral breast masses ranging from 0.5 to 5 cm in size

**Fig. 3:**
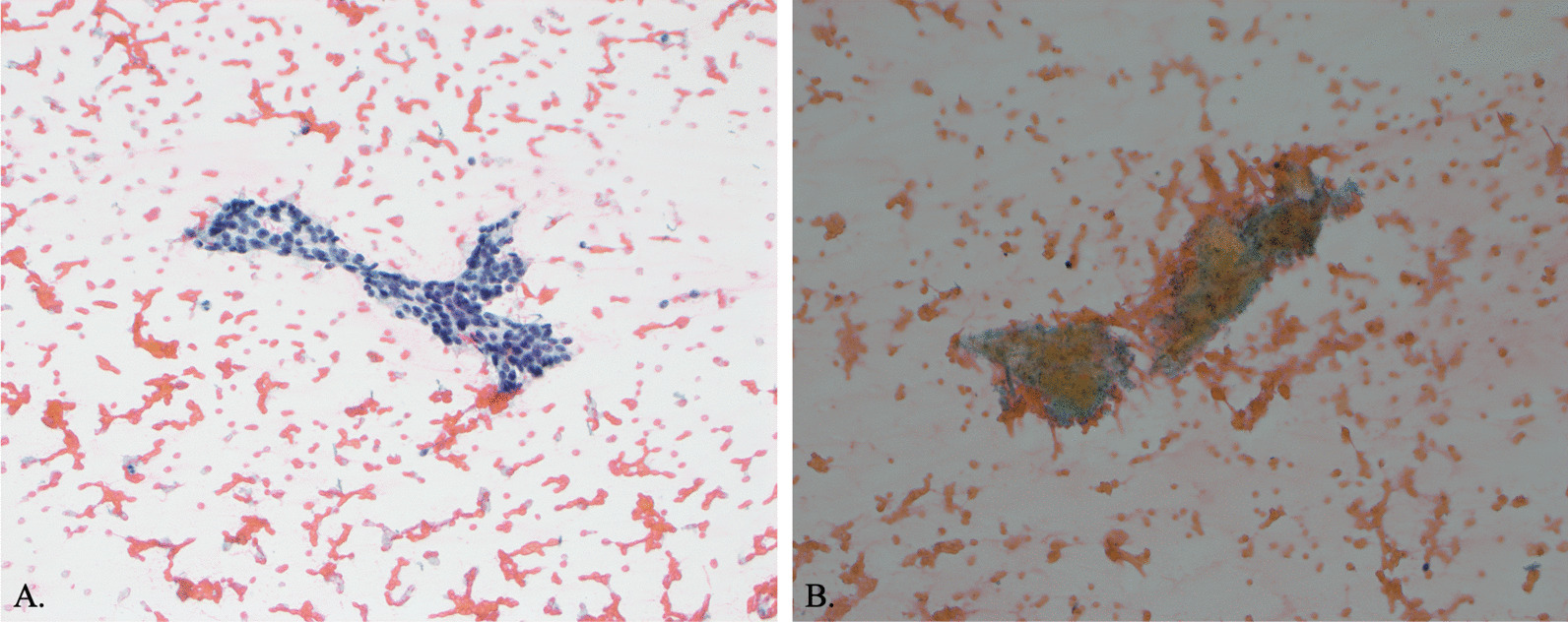
Fine needle aspiration with papanicolaou stain, x20. The aspirates were paucicellular and revealed staghorn shaped cohesive clusters of benign ductal cells. Myoepithelial cells were present in association with the ductal clusters and as bare bipolar cells in the background (**a**). Rare fragments of dense sclerotic stroma were present (**b**)

Magnetic resonance imaging of the breasts (Fig. 2) showed innumerable, bilateral breast masses ranging in size from 0.5 to 4 cm, and two fine needle biopsies cytomorphology supported a diagnosis of fibroadenoma (Fig. 3). Alternative diagnoses including phyllodes tumor were considered but not seen on biopsy. This patient did not meet criteria for testing under NCCN guidelines and suspicion for underlying hereditary cancer risk was low. However, a custom 100 gene hereditary cancer panel was ordered (Supplementary Material), for genes such as ATM, BRCA1, BRCA2, and PTEN due to the rarity of early onset, bilateral, and multiple fibroadenomas with limited family history.

Genetic testing identified a pathogenic mutation in the *PTEN* gene consistent with *PTEN* hamartoma tumor syndrome. Subsequent genetic testing of both parents was negative, consistent with a de novo mutation in this patient, which occurs in 11–48% of *PTEN* mutation carriers [10].

After discussion of management options, including excisional biopsy of the largest mass, observation, high risk screening with breast MRI, or risk reducing bilateral mastectomies, this patient opted for surgical risk reduction with bilateral mastectomies and reconstruction in the near future. This decision was based on the high lifetime risk of breast cancer, difficulty with surveillance due to the presence of innumerable masses, and patient’s symptoms.

## Discussion and conclusions

Although solitary fibroadenomas are very common and benign, the presence of multiple, bilateral fibroadenomas in a young patient should raise suspicion for alternative pathology, which can be assessed with genetic testing. Although this is a single case report, this is the first report of genetic testing in this rare presentation of fibroadenomas. Identification of a PTEN mutation allowed for appropriate cancer screening and risk reducing measures for this patient. Further studies are needed to evaluate the utility and cost-effectiveness of genetic testing for all patients with numerous and bilateral fibroadenomas. The frequency of genetic mutations in these patients is currently unknown, highlighting the importance of genetic testing in patients with unusual presentations of fibroadenomas.

## Supplementary Information

**Additional file 1**. Custom 100 Gene Hereditary Cancer Panel

## Data Availability

All data generated or analyzed during this study are included in this published article.

## Abbreviations

MRI: Magnetic resonance imaging
NCCN: National Comprehensive Cancer Network
PTEN: Phosphatase and tensin homolog
