# Supplementary material for: Early onset, multiple, bilateral fibroadenomas of the breast: a case report
Source: BMC Womens Health. 2021 Apr 21;21:170. doi: 10.1186/s12905-021-01311-7 (PMC8061223; doi:10.1186/s12905-021-01311-7)
Supplement: Supplementary file 1 — Additional file 1. Custom 100 Gene Hereditary Cancer Panel [file 12905_2021_1311_MOESM1_ESM.docx]

Germline genetic testing was performed on the following 100 genes:

ABRAXAS1, AIP, AKT1, ALK, APC, ATM, ATR, AXIN2, BAP1, BARD1, BLM, BMPR1A, BRCA1, BRCA2, BRIP1, CASR, CDC73, CDH1, CDK4, CDKN1B, CDKN2A, CHEK2, CTNNA1, DICER1, DIS3L2, EGFR, EPCAM, ERBB2, FANCA, FANCB, FANCC, FANCD2, FANCE, FANCF, FANCG, FANCI, FANCL, FANCM, FH, FLCN, GALNT12, GEN1, GREM1, HOXB13, KIF1B, KIT, MAX, MEN1, MET, MITF, MLH1, MLH3, MRE11, MSH2, MSH3, MSH6, MUTYH, NBN, NF1, NF2, NTHL1, PALB2, PALLD, PDGFRA, PHOX2B, PIK3CA, PMS2, POLD1, POLE, POT1, PRKAR1A, PRSS1, PTCH1, PTEN, RAD50, RAD51C, RAD51D, RB1, RECQL, RET, RINT1, RNF43, RPS20, SDHA, SDHAF2, SDHB, SDHC, SDHD, SLX4, SMAD4, SMARCA4, SMARCB1, SMARCE1, STK11, SUFU, TMEM127, TP53, TSC1, TSC2, VHL
